# The Possible Role of Neural Cell Apoptosis in Multiple Sclerosis

**DOI:** 10.3390/ijms23147584

**Published:** 2022-07-08

**Authors:** Peter G. E. Kennedy, Woro George, Xiaoli Yu

**Affiliations:** 1 Institute of Infection, Immunity and Inflammation, University of Glasgow, Glasgow G12 8QQ, UK; peter.kennedy@glasgow.ac.uk; 2 Department of Neurosurgery, University of Colorado Anschutz Medical Campus, Aurora, CO 80045, USA; woro.george@cuanschutz.edu

**Keywords:** multiple sclerosis, neurodegeneration, oligodendrocyte, astrocyte, microglia, neuron, axonal loss, apoptosis, demyelination

## Abstract

The etiology of multiple sclerosis (MS), a demyelinating disease affecting the central nervous system (CNS), remains obscure. Although apoptosis of oligodendrocytes and neurons has been observed in MS lesions, the contribution of this cell death process to disease pathogenesis remains controversial. It is usually considered that MS-associated demyelination and axonal degeneration result from neuroinflammation and an autoimmune process targeting myelin proteins. However, experimental data indicate that oligodendrocyte and/or neuronal cell death may indeed precede the development of inflammation and autoimmunity. These findings raise the question as to whether neural cell apoptosis is the key event initiating and/or driving the pathological cascade, leading to clinical functional deficits in MS. Similarly, regarding axonal damage, a key pathological feature of MS lesions, the roles of inflammation-independent and cell autonomous neuronal processes need to be further explored. While oligodendrocyte and neuronal loss in MS may not necessarily be mutually exclusive, particular attention should be given to the role of neuronal apoptosis in the development of axonal loss. If proven, MS could be viewed primarily as a neurodegenerative disease accompanied by a secondary neuroinflammatory and autoimmune process.

## 1. Introduction

**Figure 1:**
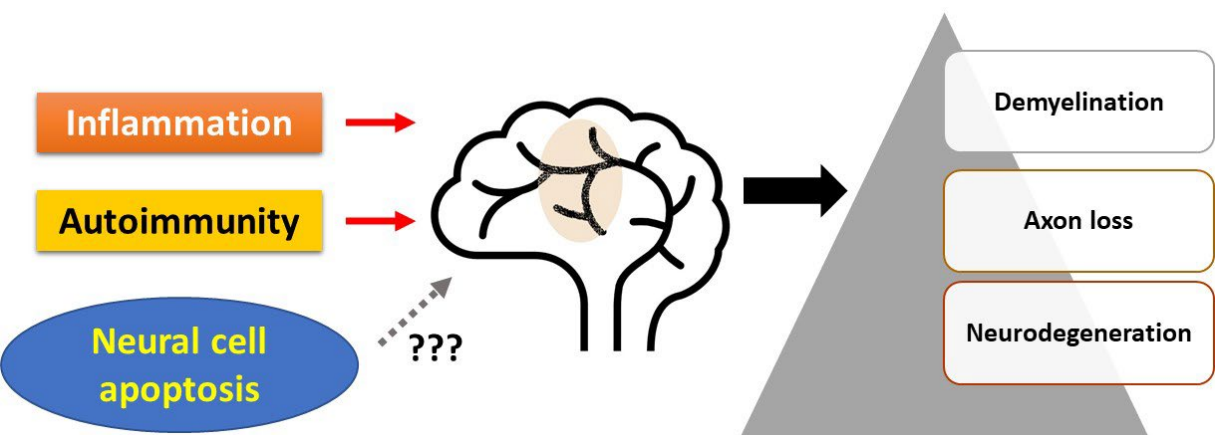
Current view of disease mechanisms of MS. It is usually considered that inflammation and autoimmunity in the CNS result in demyelination, axonal loss, and neurodegeneration. However, apoptosis of neural cells (oligodendrocytes and neurons) has been observed in MS lesions, and the contribution of this cell death process to disease pathogenesis remains to be determined.

Multiple sclerosis (MS) is an inflammatory, demyelinating, and neurodegenerative disease of the CNS. Concomitant genetic and environmental factors are important in disease pathogenesis. Genome-wide association studies indicate that common variation in the regulatory regions of immune genes drives susceptibility to MS [1]. Recently, environmental risk factors, especially EBV, have been reported as a trigger for the development of MS [2,3]. The pathologic hallmark of MS consists of focal demyelinated plaques within the CNS, with variable degrees of inflammation, gliosis, and neurodegeneration [4]. Axonal loss, a well-demonstrated pathological feature of MS [5], is often regarded as one of the pathological substrates of permanent neurological disability, as well as the presence of white matter scar formation following demyelination. Neuron vulnerability is relevant to neurodegeneration and lesion progression [6], and acute axonal damage is prominent at the earliest stages of the disease [5,7,8]. It is usually considered that inflammation and autoimmunity in the CNS result in demyelination, axonal loss, and neurodegeneration. However, apoptosis of neural cells (oligodendrocytes and neurons) has been observed in MS lesions, and the contribution of this cell death process to disease pathogenesis remains to be determined (Figure 1).

A body of literature supports the notion that apoptosis in both glia and neurons in the CNS may play a critical role in MS disease pathogenesis. In this review, we present evidence supporting apoptosis in oligodendrocytes, astrocytes, and neurons, the role of active microglia in engulfing apoptotic cells, and the relationship between apoptosis and demyelination.

## 2. In MS, Demyelination and Neuron Loss May Possibly Occur Simultaneously

Demyelination of cerebral white matter is the pathological hallmark of MS. The classical notion of demyelination is that the disease process in MS causes destruction of oligodendrocytes, which make myelin in the CNS, and this leads to demyelination [9]. The actual pathological sequence of events that lead to oligodendrocyte damage or loss is currently unclear, but it seems very likely that there is a form of immune-mediated attack on these cells. A range of toxic and/or immunological insults can damage oligodendrocytes and ensure cell death. The oligodendrocyte death causes destruction of the myelin sheath, which covers the axon, with secondary axonal degeneration.

Demyelination is thought to drive neuronal degeneration and permanent neurological disability in individuals with multiple sclerosis [10]. However, it is theoretically possible that the process of oligodendrocyte loss and axonal damage may take place simultaneously, with the latter occurring pathologically as an early event in the disease. It may be argued that axonal damage is the pivotal pathological event in MS, one that may lead to secondary rather than primary demyelination. Recent studies suggest that cerebral white matter demyelination and cortical neuronal degeneration can be independent events in the disease [10]. It is fully recognized that acute axonal damage and neuronal loss are very different pathological hallmarks that are not necessarily related to each other. However, acute axonal damage is an important feature of human demyelination, and it is still possible that actual neuronal loss may occur concomitantly with axonal loss. Multiple studies have demonstrated that at disease onset, there is already widespread neuronal and axonal dysfunction seen in MS patients [11,12,13,14]. Therefore, it is important to ascertain whether significant neuronal loss as opposed to acute axonal damage occurs in early MS lesion/disease stages. However, neuronal death at the early stage of MS does not necessarily mean that neuronal loss occurs concurrently with demyelination since it is possible that the neuronal death may occur only when neurons are within or spatially close to inflammatory regions. Utilizing post-mortem brains of patients with MS, Trapp et al. concluded that demyelination and neuronal degeneration can be independent events [10]; thus, it is conceivable to say that there is neuronal loss outside of demyelinated lesions in MS, but this may still be due to the demyelinating process in other locations. Loss of axonal function with myelin damage has the neurophysiological consequences of nerve conduction block, failure to propagate a train of nervous impulses, and conduction sensitivity to temperature alterations [15], which explain, at least in part, the multifocal functional deficits in MS patients. Functional deficits reflect the widespread distribution of myelinated neurons in the white matter of the CNS.

## 3. Apoptosis/Programmed Cell Death

Apoptosis is the process of programmed cell death, a ubiquitous process which can be triggered by a variety of factors, including removal of unwanted cells and tissues during normal development, viral infections [16], and other disruptors of normal cellular functions [17,18]. Apoptosis can be recognized histologically by the presence of characteristic abnormalities such as membrane blebbing and fragmentation of DNA [18], and by identifying caspase activity that results in DNA cleavage, leading to cell death [19], as opposed to cellular necrosis. Applications of fluorescently labeled Annexin V, which binds to phosphatidylserine that is exposed on the outside of apoptotic cells in the early stage, are popular assays [20].

Apoptosis may occur via an extrinsic or an intrinsic pathway. In the former case, the apoptotic pathway occurs via a ‘death receptor’ (related to tumor necrosis factor (TNF)), the Fas ligand (FasL). FasL signaling leads to the formation of a Fas-associated domain with FasL, resulting in the activation of caspase-3 through cleavage of procaspase-8 [19,21,22]. In contrast, the intrinsic apoptotic pathway occurs in the cell’s mitochondrion. This process is initiated by intracellular signals, following which cytochrome c is released from the mitochondrial membrane into the cell’s cytoplasm. Once in the cytoplasm, cytochrome c forms a complex that recruits and activates caspase-9, which in turn activates caspase-3 and subsequent DNA cleavage [22,23]. A detailed review of recent concepts in other programmed cell death pathways, such as necroptosis, can be found elsewhere [24]. In brief, necroptosis is an alternative cellular death pathway that has features of both apoptosis and necrosis that is activated in conditions where the capasase-8-dependent apoptotic pathway is unable to be activated [25]. In MS, there is an upregulation of tumor neurosis factor α (TNFα) in active lesions and CSF of MS patients [26,27]. Ofengeim et al. provide evidence that TNFα can induce necroptosis in MS [28].

## 4. Oligodendrocyte Apoptosis and Demyelination

### 4.1. Myelin-Directed Autoimmunity Triggers Oligodendrocyte Apoptosis in Animal Models of MS

In experimental autoimmune encephalomyelitis (EAE), an animal model for multiple sclerosis, immunization with myelin antigens leads to demyelination and paralysis. Most studies of oligodendrocyte apoptosis during the process of demyelination have been in EAE. Hisahara et al. demonstrated that caspase-11 mediates oligodendrocyte cell death as well as the pathogenesis of autoimmune-mediated demyelination in knockout mice. Murine caspase-11, a member of the caspase-1 subfamily, is required for caspase-1-induced apoptosis and IL-1β secretion. They show that both caspase-11 and activated caspase-3 were expressed in oligodendrocytes in spinal cord EAE lesions. Considering this, they concluded that oligodendrocyte death is mediated by an apoptotic pathway that involves caspases-11 and 3. The ramification of this pathway is the demyelination observed in EAE [29]. Hövelmeyer et al. have shown that apoptosis of oligodendrocytes via Fas and TNF-R1 is a key event in the induction of EAE [30]. Another group demonstrated that in C57 BL/6 EAE mice, apoptosis in the spinal cord occurred as early as three days post-immunization (p.i), and the phytochemical curcumin significantly reduced the number of apoptotic cells and inhibited the upregulation of cyt-c, caspase-9, and caspase-3 at seven days p.i. in the EAE mice [31]. 

### 4.2. Theiler’s Murine Encephalomyelitis Virus-Induced Demyelinating Disease and Other Virus-Induced Animal Models

Oligodendrocyte apoptosis has also been studied in animal models other than EAE. For example, oligodendrocyte apoptosis has been produced in an adult rat brain model using a lentivirus. The latter virus was used to express experimentally inducible caspase-9 (iCP9) cDNA under transcriptional control of the promoter for myelin basic protein, which was specific to oligodendrocytes [32]. This induced oligodendrocyte apoptosis culminated in both rapid demyelination and localized microglial activation in the absence of peripheral immune cell infiltration. Furthermore, the MBP-iCP9-induced oligodendrocyte apoptosis compromised the rate and extent of adult remyelination, and the failure of remyelination correlated with a truncated proliferative response of oligodendrocyte progenitor cells. The significance of this finding was that oligodendrocyte apoptosis clearly and directly resulted in rapid demyelination. Caprariello et al. later reported that apoptosis of oligodendrocytes during early development delays myelination and impairs subsequent responses to demyelination [33], providing direct evidence that in animal models, oligodendrocyte apoptosis leads to demyelination. Using MBP-iCP9 transgenic mice to examine demyelination and remyelination in the optic nerve, Pajoohesh-Ganji et al. showed that targeted oligodendrocyte apoptosis in the optic nerve leads to persistent demyelination [34]. In the study, oligodendrocyte death was seen within 2–3 days and demyelination was detectable along the nerve in discrete patches one week later.

Astrocyte apoptosis has also been reported in animal models of virus-induced demyelination. Using Theiler’s murine encephalomyelitis virus (TMEV) animal model of MS, a chronic, immune-mediated demyelination, Palma et al. demonstrated the presence of apoptotic glial fibrillary acidic protein (GFAP)-positive cells in the white matter of TMEV-infected mice. Apoptosis of activated astrocytes was found to be mediated by virus-specific T helper cells via a Fas–FasL interaction [35]. This study showed apoptosis in a different glial cell, so possibly the apoptotic cell type may be different in virally induced demyelination. Apoptosis accompanied by demyelination in CNS white matter was also detected in another virus infection, experimental Usutu virus infection of suckling mice [36], although inflammatory infiltrates were scarce in this model.

### 4.3. Oligodendrocyte Apoptosis in Patients with MS

Oligodendrocyte apoptosis was reported to be the earliest pathological change observed in new white matter lesions [37]. The authors examined the brains of 12 patients with relapsing and remitting MS. Using conventional histology and immunohistochemical methods, they reported that the earliest change observed in the lesions was widespread oligodendrocyte apoptosis in tissue with normal appearing T cells, macrophages, activated microglia, reactive astrocytes, and neurons. Extensive oligodendrocyte apoptosis, along with microglial activation, was detected in myelinated tissue containing few or no lymphocytes or myelin phagocytes. No direct evidence of the underlying cause of the oligodendrocyte apoptosis was determined, but the findings did suggest that there could be some form of novel process which underlies new lesion formation in MS [37]. It would be important, however, to convincingly show that oligodendrocytes expressing activated caspase-3 occur in MS lesions which, to our knowledge, has not yet been achieved. Furthermore, early loss of oligodendrocytes was found to be a prominent feature in tissues bordering rapidly expanding MS lesions [38]. Another intriguing question that needs to be addressed is whether, and how, oligodendrocyte apoptosis differs in lesional versus ‘normal appearing’ areas. The neuropathological work of Lucchinetti [39] is relevant to the current discussion. Lucchinetti et al. identified four fundamentally different patterns of demyelination in MS brains. The patterns of types III and IV were very suggestive of a primary oligodendrocyte dystrophy, reminiscent of virus- or toxin-induced demyelination. Interestingly, in the current context, 14–37% of pattern III brains showed evidence of oligodendrocyte apoptosis based on histological analysis [39].

An additional indication that apoptotic mechanisms may operate in MS came from Hagman et al.’s work, in which blood levels of sFas were found to be increased in patients with MS. The MS patients had deteriorating disability scores and increasing hypointense lesions on magnetic resonance imaging (MRI) [40]. Rinta et al. reported that the Fas-ligand was increased in the sera of RRMS patients during relapse [41]. In addition, the extrinsic pathway adaptor, FADD, was shown to be upregulated in RRMS individuals [42]. The switch from clinically isolated syndrome (CIS) to MS, in a cohort consisting of a limited number of patients, was associated with a different profile of apoptosis-related gene transcription in comparison with no converting CIS patients [43].

In a detailed review by Guire et al., the authors considered the process of apoptosis to be present within MS lesions, and death receptor signaling, a process capable of triggering a pathway leading to apoptosis, was noted to be important in CNS demyelination [27]. In another review of this subject, Artemiadis et al. noted that apoptosis of oligodendrocytes is an early event in MS that precedes the formation of the demyelinated plaque and post-translational modifications of myelin basic protein, characteristic processes of normal-appearing white matter, in MS [44]. They emphasized the importance of studying such events at a very early stage of the disease as well as at sequential points. Whether oligodendrocyte apoptosis occurs as an early feature in new MS lesions, or if this occurs in a subgroup of MS patients, is still a matter of debate. The classical notion of MS pathology is that myelin and oligodendrocyte loss may happen simultaneously, or that oligodendrocyte loss may be secondary to myelin destruction.

## 5. Apoptosis in Neurons in Demyelinating Diseases

### 5.1. In MS, Neuronal Death Is Prominent and Correlates with Disease Progression

Axonal loss is a key pathological feature of MS demyelinating lesions, and axonal transection is seen in active and chronic active lesions from patients with durations of clinical disease ranging from 2 weeks to 27 years [5]. In addition, immunological attack with the membrane attack complex (MAC) of complement causes severe demyelination that is associated with acute axonal injury [45] and grey matter pathology that correlates with physical disabilities and cognitive impairment [46,47,48,49]. Furthermore, it has been recently demonstrated at the molecular levels that neuron vulnerability is relevant to neurodegeneration and lesion progression [6]. Evidence that axonal loss is prominent at the earliest stages of the disease [5,7] indicates that this loss may be an early feature of the demyelinating process. Activation of necroptosis signaling in cortical neurons of secondary progressive MS patients supports that neuron loss plays a significant role in disease pathogenesis [50]

### 5.2. Neuron Apoptosis in MS

Apoptosis mediates the precise and programmed death of neurons. It is a physiologically important process in neurogenesis during maturation of the CNS. However, premature apoptosis and/or an aberration in apoptosis regulation is implicated in the pathogenesis of neurodegeneration. This is a multifaceted process that leads to various chronic disease states, such as Alzheimer’s, Parkinson’s, and Huntington’s diseases, amyotrophic lateral sclerosis, spinal muscular atrophy, and diabetic encephalopathy [51]. While the mechanisms of demyelination and axonal loss/neuron death are yet to be established, a body of literature supports that apoptosis in glia and neurons in the CNS may play critical roles in MS disease pathogenesis. While MS clearly differs in several respects from classic neurodegenerative diseases, because it has such an important neuroinflammatory component, nevertheless, because of the clinical results of MS on the CNS, we do think it can be conceptualized as a type of neurodegenerative disease, especially in its final stages. Furthermore, it is possible that oligodendrocyte precursor cells (OPC) actively contribute to the inflammation, which further contributes, directly and indirectly, to neurodegeneration [52]. Many other CNS resident cells can also contribute to this neuroinflammation, and this in turn promotes neurodegeneration. As such, it is reasonable to characterize MS as both a neuroinflammatory and neurodegenerative disease.

A study by Alcázar et al. investigated the effects of cerebrospinal fluid (CSF)-soluble factors from MS patients on neurons in tissue culture [53]. They found that CSF from progressive MS patients termed as “aggressive”, meaning those with poor recovery following acute relapses, induced axonal damage and neuronal apoptosis. The induced neuronal damage was not related to either blood–brain barrier (BBB) dysfunction or the IgG index. In contrast, when the CSF from MS patients deemed “non-aggressive”, meaning those with a relapsing–remitting course with a good recovery after relapses, was similarly tested, they did not induce neuronal damage. The authors, therefore, speculated that it was the neuronal damage that might account for the various types of functional deficit seen in MS patients. An early study of an EAE rat model of MS demonstrated the presence of acute neuronal apoptosis [54]. Furthermore, caspase inhibitors are shown to be protective against neuronal apoptosis induced by MS CSF [55].

Lisak et al. reported somewhat similar findings in which B cells, obtained from the blood of MS patients with relapsing–remitting disease, but not normal controls, secreted a factor or factors that induced apoptosis in cultured rat neurons [56]. However, the authors concluded that neuronal cytotoxicity was not mediated by complement and was also independent of either immunoglobulin or multiple cytokines. Clearly, there is an intriguing link between the serum, CSF of MS patients, and neuronal apoptosis. 

### 5.3. Other Clinical Evidence for the Possible Role of Neuronal Damage in MS

Other lines of clinical evidence provide indirect support for a process other than oligodendrocytes being a critical determinant of demyelination in MS. For example, neuropathological studies indicate that in MS, grey matter pathology, due primarily to neuronal loss, can be observed in the absence of inflammation [37]. Further, retinal cells, though they do not contain myelin, have nevertheless been shown to atrophy in MS [57]. This latter observation strongly suggests that neural cell loss in MS does not necessarily require the presence of a demyelinating process.

## 6. Neuronal Apoptosis and Microglia

Microglia are the resident phagocytic cells of the CNS and are functionally and morphologically similar to the monocyte-derived macrophages [58]. Microglia have various active states with marked diversity [59] in functions, which include phagocytosis, antigen presentation, cytokine production, and oxidative bursts [58]. In active MS, most of the lesions are composed of microglia/macrophages with a microglia predominance [60,61]. While inactive lesions have substantially fewer microglia, active demyelination in MS is associated with aggregation of activated microglia. As noted above, Caprariello et al. found that demyelination triggered an expeditious and potent activation of microglia [32]. These activated microglia can release various cytotoxic molecules, including TNFα, reactive oxygen species (ROS), and various interleukins, including 1β and 6, that can mobilize and induce apoptotic cascades within neurons [62].

TNFα expression patterns are high in active MS lesions [26,27], and CSF TNF was found to be increased in MS patients at the time of diagnosis [63]. TNFα is an intriguing cytokine in that depending on the receptor it uses for signaling (TNF receptors 1 and 2), it either can promote neuronal death or survival [64]. Guadagno et al. showed that activated microglia-derived TNFα induced apoptosis in neuronal precursor cells (NPC) [65]. This group also found that soluble factors from these microglia induced apoptosis in NPCs. Other activated microglia products such as ROS and NOS may lead to oxidative damage [66], and eventually neuronal death. Cserep et al. demonstrate that microglial and neuronal function are intimately intertwined [67,68]. Anderson et al. show that interactions between apoptotic neurons remodel microglia to states that are less homeostatic [69]. In active MS lesions, expression of P2RY12, a homeostatic marker on microglia [66], on activated microglia is reduced compared to normal controls which had intermediate pro-inflammatory and P2RY12 phenotypes [61]. Zrzavy et al. concluded that microglia in active MS lesions tended to have a loss of P2RY12 homeostatic expression [61]. This view is consistent with the work of Butovsky et al., which showed the same pattern in other neurodegenerative diseases [66]. Anderson et al. describe the heterogeneity of microglia in postnatal retina, and one of the many conclusions that they made was that neuronal apoptosis drives remodeling states of microglia to less homeostatic states [69]. Anderson et al. bring forth an interesting notion, the notion of neuronal apoptosis and microglial states being interlinked, and thus it is plausible to speculate that neuronal apoptosis potentially reinforces and amplifies microglia destruction. It is, therefore, also conceivable that perhaps it is the neuronal apoptosis itself that is critical in the pathogenesis of MS.

## 7. Implications of Apoptosis in Demyelination and MS Disease Pathogenesis

### 7.1. Possible Roles of Apoptosis in Demyelination and Disease Activities

It has been clearly demonstrated that oligodendrocyte apoptosis occurs early in the demyelinating process in both MS lesions and animal models. Although axonal injury correlates with immune-mediated demyelination in MS [45], it is yet to be established whether neuronal apoptosis occurs independently of demyelination. Identification of the underlying pathological process in MS is important because it enhances our understanding of the disease pathogenesis. The presence of apoptosis provides indirect evidence of a viral etiology, as virus infection can induce apoptosis, or other unknown mechanisms yet to be uncovered. The possible viral etiology of MS has been discussed in detail elsewhere [70] and is outside the scope of the present discussion, but a strong contender for the viral hypothesis of MS pathogenesis is Epstein-Barr virus (EBV). There are several direct and indirect lines of evidence of EBV’s involvement in the pathogenesis of MS [71,72].

The significance of glial cell apoptosis in demyelination is still unclear, and the interpretation of such data is speculative. In the case of oligodendrocyte apoptosis, however, it is not difficult to assume that this will inevitably lead to oligodendrocyte loss, resulting in primary demyelination of the disease. Following myelin loss, axonal injury would then surely follow. While this interpretation follows classical lines, it is also important to consider the possible role of neuronal apoptosis in this disease. It has already been noted that axonal loss is an early event in the development of both active and chronic active MS lesions [5]. Therefore, it is conceivable that a potential pathological process in MS could begin with neuronal apoptosis, leading to primary axonal injury and secondary demyelination. Indeed, neuronal apoptosis has been implicated in the pathogenesis of neurodegenerative diseases [51].

This latter concept has been articulated in detail by Tsunoda and Fujinami, and in our view, it is a credible viewpoint. In this so-called ‘Inside-Out model’ of demyelination, a different pathogenetic scenario is envisaged in which the initial event in MS demyelination is neuronal apoptosis. This process leads to axonal injury, the process of which causes oligodendrocyte-axon disruption, and this in turn induces microglial activation, which could result in further oligodendrocyte apoptosis as well as secondary demyelination [73].

Based on the cumulative evidence generated in the last two decades, it is possible that both oligodendrocyte and neuronal apoptosis take place simultaneously, and the two processes need not be mutually exclusive. It is plausible that the key pathological process is neuronal apoptosis and that the observed demyelination may be a consequence of this rather than the cause. A practical issue is that conceivably, the apoptotic cells in MS lesions are constantly being removed by microglia, a process that might explain why pathological studies of MS lesions rarely reveal them. While it is generally assumed that the acute inflammatory white matter lesions provide the pathological substrate of the acute clinical features of relapsing–remitting MS, and the axonal loss accounts for the irreversible neurological deficits, this is not the only possible explanation of the functional deficits in MS. Under this view, at least some of the demyelination and neurological symptoms might be a consequence of neuronal apoptosis, and further, the functional CNS deficits may be due to neuronal death rather than demyelination per se. If the apoptotic oligodendrocytes and neurons are engulfed by phagocytic microglia, then this itself may be anti-inflammatory, since it has been demonstrated that phagocytosis of apoptotic cells has anti-inflammatory effects [16,74]. The consequences of this are unclear because if the inflammation is ‘beneficial’, then inhibiting it would be deleterious, but if the inflammation is ‘harmful’, then inhibiting it would be advantageous [16].

There are at least four different potential avenues for future investigations to unravel the issues of apoptosis and demyelination in MS disease mechanisms. The first is comprehensive studies of mouse knockouts lacking key apoptosis genes, and/or transgenic mice that express anti-apoptotic genes, that should result in reduced or absent demyelination in different experimental models of demyelination, including but not confined to EAE. Secondly, whole exome sequencing of MS patients could be screened for non-functioning mutations of key apoptotic genes. Thirdly, advanced MRI scans of MS patients carried out very early in the disease, as well as sequentially, could be scrutinized in detail for evidence of early neuronal loss. Lastly, brain specimens from deceased MS patients could be examined in detail for evidence of apoptotic staining in all glial cell types using double staining methodology with both cellular and apoptotic markers.

### 7.2. Failure of Remyelination 

There is evidence that early in the disease, demyelinated plaques can remyelinate, but this event is often incomplete, variable among patients, and not sufficient to overcome disease progression [75,76,77]. Although it is still unknown why spontaneous remyelination fails over time in MS, the cause probably involves a depletion of oligodendrocyte precursor cells (OPCs) as well as their decreased ability to migrate to sites of injury and differentiate into myelinating oligodendrocytes [76,78]. Perhaps, the key player in this process is the oligodendrocyte, and apoptosis in OPCs may prevent remyelination.

## 8. Conclusions

**Figure 2:**
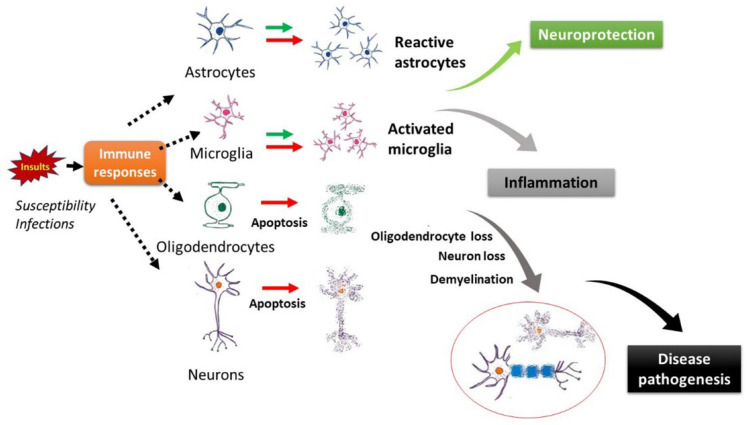
Schematic representation of the pathological process in MS. Infections in genetically susceptible individuals trigger responses in CNS cells, resulting in activation of astrocytes/microglia and apoptosis in oligodendrocytes/neurons. The activated glia can be protective or detrimental, which produce anti/proinflammatory cytokines in the CNS. The damaged oligodendrocytes and neurons result in demyelination and neurodegeneration, contributing to disease pathogenesis.

Our hypothesis is that in MS infections in genetically susceptible individuals trigger responses in CNS cells, resulting in activation of astrocytes/microglia and apoptosis in oligodendrocytes/neurons. The activated glia can be protective or detrimental and can produce anti/proinflammatory cytokines in the CNS. The damaged oligodendrocytes and neurons result in demyelination and neurodegeneration, contributing to disease pathogenesis (Figure 2). While there is abundant evidence for the existence of neuronal cell apoptosis in MS, the relative pathogenic importance of oligodendrocyte and axonal damage/neuronal apoptosis in MS remains unclear. Damage to both axons and oligodendrocytes are the early neuropathological features of the disease. The conventional view is to equate apoptosis in the myelin-forming oligodendrocyte with the classical demyelinating lesions, but there is also increasing evidence from neuropathological, clinical epidemiological, and experimental studies for the pathogenic importance of neuronal apoptosis as being a key initial event which may lead to secondary demyelination and functional deficits. Axonal damage correlates with the degree of neuroinflammation in MS and is a prominent feature of the disease. If this proves to be the case in most, if not all, MS disease phenotypes, this will have considerable significance for both understanding the underlying pathogenesis of MS and potential therapies that could be directed primarily at restoring neuronal function, including fast axonal transport.

## References

[1] Patsopoulos N., Barcellos L.F., Hintzen R.Q., Schaefer C., Van Duijn C.M., Noble J.A., Raj T., Gourraud P.-A., Stranger B., Oksenberg J. (2013). Fine-Mapping the Genetic Association of the Major Histocompatibility Complex in Multiple Sclerosis: HLA and Non-HLA Effects. PLoS Genet..

[2] Bjornevik K., Cortese M., Healy B.C., Kuhle J., Mina M.J., Leng Y., Elledge S.J., Niebuhr D.W., Scher A.I., Munger K.L. (2022). Longitudinal analysis reveals high prevalence of Epstein-Barr virus associated with multiple sclerosis. Science.

[3] Lanz T.V., Brewer R.C., Ho P.P., Moon J.-S., Jude K.M., Fernandez D., Fernandes R.A., Gomez A.M., Nadj G.-S., Bartley C.M. (2022). Clonally expanded B cells in multiple sclerosis bind EBV EBNA1 and GlialCAM. Nature.

[4] Popescu B.F., Pirko I., Lucchinetti C.F. (2013). Pathology of multiple sclerosis: Where do we stand?. Continuum.

[5] Trapp B.D., Peterson J., Ransohoff R.M., Rudick R., Mörk S., Bö L. (1998). Axonal Transection in the Lesions of Multiple Sclerosis. N. Engl. J. Med..

[6] Schirmer L., Velmeshev D., Holmqvist S., Kaufmann M., Werneburg S., Jung D., Vistnes S., Stockley J.H., Young A., Steindel M. (2019). Neuronal vulnerability and multilineage diversity in multiple sclerosis. Nature.

[7] Ferguson B., Matyszak M.K., Esiri M.M., Perry V.H. (1997). Axonal damage in acute multiple sclerosis lesions. Brain.

[8] Cree B.A., Hollenbach J.A., Bove R., Kirkish G., Sacco S., Caverzasi E., Bischof A., Gundel T., Zhu A.H., San Francisco MS-EPIC Team of University of California (2019). Silent progression in disease activity-free relapsing multiple sclerosis. Ann. Neurol..

[9] Stassart R.M., Möbius W., Nave K.-A., Edgar J.M. (2018). The Axon-Myelin Unit in Development and Degenerative Disease. Front. Neurosci..

[10] Trapp B.D., Vignos M., Dudman J., Chang A., Fisher E., Staugaitis S.M., Battapady H., Mork S., Ontaneda D., E Jones S. (2018). Cortical neuronal densities and cerebral white matter demyelination in multiple sclerosis: A retrospective study. Lancet Neurol..

[11] Calabrese M., Atzori M., Bernardi V., Morra A., Romualdi C., Rinaldi L., McAuliffe M.J.M., Barachino L., Perini P., Fischl B. (2007). Cortical atrophy is relevant in multiple sclerosis at clinical onset. J. Neurol..

[12] Filippi M., Bozzali M., Rovaris M., Gonen O., Kesavadas C., Ghezzi A., Martinelli V., Grossman R.I., Scotti G., Comi G. (2003). Evidence for widespread axonal damage at the earliest clinical stage of multiple sclerosis. Brain.

[13] Kapeller P., McLean M.A., Griffin C.M., Chard D., Parker G.J., Barker G.J., Thompson A.J., Miller D.H. (2001). Preliminary evidence for neuronal damage in cortical grey matter and normal appearing white matter in short duration relapsing-remitting multiple sclerosis: A quantitative MR spectroscopic imaging study. J. Neurol..

[14] Kurnellas M., Donahue K., Elkabes S. (2007). Mechanisms of neuronal damage in multiple sclerosis and its animal models: Role of calcium pumps and exchangers. Biochem. Soc. Trans..

[15] Compston A., Coles A. (2008). Multiple sclerosis. Lancet.

[16] Kennedy P.G. (2015). Viruses, apoptosis, and neuroinflammation—A double-edged sword. J. Neurovirol..

[17] Kerr J.F.R., Wyllie A.H., Currie A.R. (1972). Apoptosis: A Basic Biological Phenomenon with Wideranging Implications in Tissue Kinetics. Br. J. Cancer.

[18] Wyllie A.H. (1997). Apoptosis: An overview. Br. Med Bull..

[19] A Thornberry N. (1997). The caspase family of cysteine proteases. Br. Med Bull..

[20] Crowley L., Marfell B.J., Scott A.P., Waterhouse N.J. (2016). Quantitation of apoptosis and necrosis by annexin V binding, propidium iodide uptake, and flow cytometry. Cold Spring Harbor Protocols..

[21] Ashkenazi A., Dixit V.M. (1998). Death Receptors: Signaling and Modulation. Science.

[22] Aubert M., Pomeranz L.E., Blaho J.A. (2006). Herpes simplex virus blocks apoptosis by precluding mitochondrial cytochrome c release independent of caspase activation in infected human epithelial cells. Apoptosis.

[23] Sun X.-M., MacFarlane M., Zhuang J., Wolf B.B., Green D.R., Cohen G.M. (1999). Distinct Caspase Cascades Are Initiated in Receptor-mediated and Chemical-induced Apoptosis. J. Biol. Chem..

[24] Bertheloot D., Latz E., Franklin B.S. (2021). Necroptosis, pyroptosis and apoptosis: An intricate game of cell death. Cell. Mol. Immunol..

[25] Dhuriya Y.K., Sharma D. (2018). Necroptosis: A regulated inflammatory mode of cell death. J. Neuroinflamm..

[26] Akassoglou K., Bauer J., Kassiotis G., Pasparakis M., Lassmann H., Kollias G., Probert L. (1998). Oligodendrocyte Apoptosis and Primary Demyelination Induced by Local TNF/p55TNF Receptor Signaling in the Central Nervous System of Transgenic Mice: Models for Multiple Sclerosis with Primary Oligodendrogliopathy. Am. J. Pathol..

[27] Guire C.M., Beyaert R., van Loo G. (2011). Death receptor signalling in central nervous system inflammation and demyelination. Trends Neurosci..

[28] Ofengeim D., Ito Y., Najafov A., Zhang Y., Shan B., DeWitt J.P., Ye J., Zhang X., Chang A., Vakifahmetoglu-Norberg H. (2015). Activation of Necroptosis in Multiple Sclerosis. Cell Rep..

[29] Hisahara S., Yuan J., Momoi T., Okano H., Miura M. (2001). Caspase-11 Mediates Oligodendrocyte Cell Death and Pathogenesis of Autoimmune-Mediated Demyelination. J. Exp. Med..

[30] Hövelmeyer N., Hao Z., Kranidioti K., Kassiotis G., Buch T., Frommer F., Von Hoch L., Kramer D., Minichiello L., Kollias G. (2005). Apoptosis of Oligodendrocytes via Fas and TNF-R1 Is a Key Event in the Induction of Experimental Autoimmune Encephalomyelitis. J. Immunol..

[31] Feng J., Tao T., Yan W., Chen C.S., Qin X. (2014). Curcumin Inhibits Mitochondrial Injury and Apoptosis from the Early Stage in EAE Mice. Oxidative Med. Cell. Longev..

[32] Caprariello A.V., Mangla S., Miller R.H., Selkirk S.M. (2012). Apoptosis of oligodendrocytes in the central nervous system results in rapid focal demyelination. Ann. Neurol..

[33] Caprariello A.V., Batt C.E., Zippe I., Romito-DiGiacomo R.R., Karl M., Miller R.H. (2015). Apoptosis of Oligodendrocytes during Early Development Delays Myelination and Impairs Subsequent Responses to Demyelination. J. Neurosci..

[34] Pajoohesh-Ganji A., Miller R.H. (2019). Targeted Oligodendrocyte Apoptosis in Optic Nerve Leads to Persistent Demyelination. Neurochem. Res..

[35] Palma J.P., Yauch R.L., Lang S., Kim B.S. (1999). Potential role of CD4+ T cell-mediated apoptosis of activated astrocytes in Theiler’s virus-induced demyelination. J. Immunol..

[36] Weissenböck H., Bakonyi T., Chvala S., Nowotny N. (2004). Experimental Usutu virus infection of suckling mice causes neuronal and glial cell apoptosis and demyelination. Acta Neuropathol..

[37] Barnett M.H., Prineas J.W. (2004). Relapsing and remitting multiple sclerosis: Pathology of the newly forming lesion. Ann. Neurol..

[38] Henderson A.P.D., Barnett M.H., Parratt J.D.E., Prineas J.W. (2009). Multiple sclerosis: Distribution of inflammatory cells in newly forming lesions. Ann. Neurol..

[39] Lucchinetti C., Brück W., Parisi J., Scheithauer B., Rodriguez M., Lassmann H. (2000). Heterogeneity of multiple sclerosis lesions: Implications for the pathogenesis of demyelination. Ann. Neurol..

[40] Hagman S., Raunio M., Rossi M., Dastidar P., Elovaara I. (2011). Disease-associated inflammatory biomarker profiles in blood in different subtypes of multiple sclerosis: Prospective clinical and MRI follow-up study. J. Neuroimmunol..

[41] Rinta S., Kuusisto H., Raunio M., Paalavuo R., Levula M., Lehtimäki T., Elovaara I. (2008). Apoptosis-related molecules in blood in multiple sclerosis. J. Neuroimmunol..

[42] Reuss R., Mistarz M., Mirau A., Kraus J., Bödeker R.-H., Oschmann P. (2014). FADD Is Upregulated in Relapsing Remitting Multiple Sclerosis. Neuroimmunomodulation.

[43] Hagman S., Kolasa M., Basnyat P., Helminen M., Kähönen M., Dastidar P., Lehtimäki T., Elovaara I. (2015). Analysis of apoptosis-related genes in patients with clinically isolated syndrome and their association with conversion to multiple sclerosis. J. Neuroimmunol..

[44] Artemiadis A.K., Anagnostouli M.C. (2010). Apoptosis of Oligodendrocytes and Post-Translational Modifications of Myelin Basic Protein in Multiple Sclerosis: Possible Role for the Early Stages of Multiple Sclerosis. Eur. Neurol..

[45] Mead R.J., Singhrao S.K., Neal J.W., Lassmann H., Morgan B.P. (2002). The Membrane Attack Complex of Complement Causes Severe Demyelination Associated with Acute Axonal Injury. J. Immunol..

[46] Damjanovic D., Valsasina P., Rocca M., Stromillo M., Gallo A., Enzinger C., Hulst H., Rovira A., Muhlert N., De Stefano N. (2016). Hippocampal and Deep Gray Matter Nuclei Atrophy Is Relevant for Explaining Cognitive Impairment in MS: A Multicenter Study. Am. J. Neuroradiol..

[47] Eshaghi A., Prados F., Brownlee W.J., Altmann D.R., Tur C., Cardoso M.J., De Angelis F., van de Pavert S.H., Cawley N., De Stefano N. (2018). Deep gray matter volume loss drives disability worsening in multiple sclerosis. Ann. Neurol..

[48] Scalfari A., Romualdi C., Nicholas R.S., Mattoscio M., Magliozzi R., Morra A., Monaco S., Muraro P.A., Calabrese M. (2018). The cortical damage, early relapses, and onset of the progressive phase in multiple sclerosis. Neurology.

[49] Andica C., Hagiwara A., Kamagata K., Yokoyama K., Shimoji K., Saito A., Takenaka Y., Nakazawa M., Hori M., Cohen-Adad J. (2019). Gray Matter Alterations in Early and Late Relapsing-Remitting Multiple Sclerosis Evaluated with Synthetic Quantitative Magnetic Resonance Imaging. Sci. Rep..

[50] Picon C., Jayaraman A., James R., Beck C., Gallego P., Witte M.E., van Horssen J., Mazarakis N.D., Reynolds R. (2021). Neuron-specific activation of necroptosis signaling in multiple sclerosis cortical grey matter. Acta Neuropathol..

[51] Okouchi M., Ekshyyan O., Maracine M., Aw T.Y. (2007). Neuronal Apoptosis in Neurodegeneration. Antioxid. Redox Signal..

[52] Psenicka M.W., Smith B.C., Tinkey R.A., Williams J.L. (2021). Connecting Neuroinflammation and Neurodegeneration in Multiple Sclerosis: Are Oligodendrocyte Precursor Cells a Nexus of Disease?. Front. Cell. Neurosci..

[53] Alcázar A., Regidor I., Masjuan J., Salinas M., Álvarez-Cermeño J. (2000). Axonal damage induced by cerebrospinal fluid from patients with relapsing-remitting multiple sclerosis. J. Neuroimmunol..

[54] Meyer R., Weissert R., Diem R., Storch M.K., De Graaf K.L., Kramer B., Bähr M. (2001). Acute Neuronal Apoptosis in a Rat Model of Multiple Sclerosis. J. Neurosci..

[55] Cid C., Álvarez-Cermeño J., Regidor I., Plaza J., Salinas M., Alcázar A. (2003). Caspase inhibitors protect against neuronal apoptosis induced by cerebrospinal fluid from multiple sclerosis patients. J. Neuroimmunol..

[56] Lisak R.P., Nedelkoska L., Benjamins J.A., Schalk D., Bealmear B., Touil H., Li R., Muirhead G., Bar-Or A. (2017). B cells from patients with multiple sclerosis induce cell death via apoptosis in neurons in vitro. J. Neuroimmunol..

[57] Green A.J., McQuaid S., Hauser S.L., Allen I.V., Lyness R. (2010). Ocular pathology in multiple sclerosis: Retinal atrophy and inflammation irrespective of disease duration. Brain.

[58] Luo C., Jian C., Liao Y., Huang Q., Wu Y., Liu X., Zou D., Wu Y. (2017). The role of microglia in multiple sclerosis. Neuropsychiatr. Dis. Treat..

[59] Xue J., Schmidt S.V., Sander J., Draffehn A., Krebs W., Quester I., De Nardo D., Gohel T.D., Emde M., Schmidleithner L. (2014). Transcriptome-based network analysis reveals a spectrum model of human macrophage activation. Immunity.

[60] Guerrero B.L., Sicotte N.L. (2020). Microglia in Multiple Sclerosis: Friend or Foe?. Front. Immunol..

[61] Zrzavy T., Hametner S., Wimmer I., Butovsky O., Weiner H.L., Lassmann H. (2017). Loss of ‘homeostatic’ microglia and patterns of their activation in active multiple sclerosis. Brain.

[62] Ciesielski-Treska J., Ulrich G., Chasserot-Golaz S., Zwiller J., Revel M.-O., Aunis D., Bader M.-F. (2001). Mechanisms Underlying Neuronal Death Induced by Chromogranin A-activated Microglia. J. Biol. Chem..

[63] Magliozzi R., Pezzini F., Pucci M., Rossi S., Facchiano F., Marastoni D., Montagnana M., Lippi G., Reynolds R., Calabrese M. (2021). Changes in Cerebrospinal Fluid Balance of TNF and TNF Receptors in Naïve Multiple Sclerosis Patients: Early Involvement in Compartmentalised Intrathecal Inflammation. Cells.

[64] Kraft A.D., McPherson C.A., Harry G.J. (2009). Heterogeneity of microglia and TNF signaling as determinants for neuronal death or survival. NeuroToxicology.

[65] Guadagno J., Xu X., Karajgikar M., Brown A., Cregan S. (2013). Microglia-derived TNFalpha induces apoptosis in neural precursor cells via transcriptional activation of the Bcl-2 family member Puma. Cell Death Dis..

[66] Butovsky O., Jedrychowski M.P., Cialic R., Krasemann S., Murugaiyan G., Fanek Z., Greco D.J., Wu P.M., Doykan C.E., Kiner O. (2014). Targeting miR-155 restores abnormal microglia and attenuates disease in SOD1 mice. Ann. Neurol..

[67] Cserép C., Pósfai B., Dénes Á. (2020). Shaping Neuronal Fate: Functional Heterogeneity of Direct Microglia-Neuron Interactions. Neuron.

[68] Cserép C., Pósfai B., Lénárt N., Fekete R., László Z.I., Lele Z., Orsolits B., Molnár G., Heindl S., Schwarcz A.D. (2020). Microglia monitor and protect neuronal function through specialized somatic purinergic junctions. Science.

[69] Anderson S.R., Roberts J.M., Ghena N., A Irvin E., Schwakopf J., Cooperstein I.B., Bosco A., Vetter M.L. (2022). Author response: Neuronal apoptosis drives remodeling states of microglia and shifts in survival pathway dependence. Elife.

[70] Gilden D.H. (2005). Infectious causes of multiple sclerosis. Lancet Neurol..

[71] Wang Z., Kennedy P.G., Dupree C., Wang M., Lee C., Pointon T., Langford T.D., Graner M.W., Yu X. (2020). Antibodies from Multiple Sclerosis Brain Identified Epstein-Barr Virus Nuclear Antigen 1 & 2 Epitopes which Are Recognized by Oligoclonal Bands. J. Neuroimmune Pharmacol..

[72] Tyler K.L. (2022). The enigmatic links between Epstein-Barr virus infection and multiple sclerosis. J. Clin. Investig..

[73] Tsunoda I., Fujinami R.S. (2002). Inside-Out versus Outside-In models for virus induced demyelination: Axonal damage triggering demyelination. Springer Semin. Immunopathol..

[74] Henson P.M., Bratton D.L. (2013). Antiinflammatory effects of apoptotic cells. J. Clin. Investig..

[75] Lassmann H., Brück W., Lucchinetti C., Rodriguez M. (1997). Remyelination in multiple sclerosis. Mult. Scler..

[76] Franklin R.J. (2002). Why does remyelination fail in multiple sclerosis?. Nat. Rev. Neurosci..

[77] Patrikios P., Stadelmann C., Kutzelnigg A., Rauschka H., Schmidbauer M., Laursen H., Sorensen P.S., Brück W., Lucchinetti C., Lassmann H. (2006). Remyelination is extensive in a subset of multiple sclerosis patients. Brain.

[78] Pluchino S., Muzio L., Imitola J., Deleidi M., Alfaro-Cervello C., Salani G., Porcheri C., Brambilla E., Cavasinni F., Bergamaschi A. (2008). Persistent inflammation alters the function of the endogenous brain stem cell compartment. Brain.

